# Implicit voice learning through discrimination outperforms explicit listen-and-memorize tasks

**DOI:** 10.1038/s41598-026-41541-z

**Published:** 2026-03-14

**Authors:** Andrea Fröhlich, Meike Ramon, Peter French, Volker Dellwo

**Affiliations:** 1 Phonetics and Speech Sciences, Department of Computational Linguistics, University of Zurich, Zurich, Switzerland; 2 Speech and Audio Group, Zurich Forensic Science Institute, Zurich, Switzerland; 3 Centre for Forensic Phonetics and Acoustics, Department of Computational Linguistics, University of Zurich, Zurich, Switzerland; 4 Applied Face Cognition Lab, Institute of Applied Data Science and Finance, Bern University of Applied Sciences, Bern, Switzerland; 5 AIR-Association for Independent Research, Zurich, Switzerland; 6 JP French International, York, England

**Keywords:** Voice learning, Implicit vs. explicit exposure, Voice memorization, Speaker identity, Voice load, Speaker similarity, Neuroscience, Psychology, Psychology

## Abstract

Voice learning primarily occurs *implicitly* in everyday situations—as an incidental by-product of other activities such as participating in conversation or listening to voices in the media. Most research on voice learning is conducted in laboratory settings, where participants are *explicitly* instructed to attend to and memorize voices for later recognition. Yet, the impact of task awareness (awareness regarding the goal of voice learning) on voice recognition performance remains poorly understood. To address this gap, we conducted a study comparing two voice-learning modalities: explicit learning, instructing participants to listen to and memorize voices for later recognition, and implicit learning, based on exposure during a voice discrimination task, without awareness of a subsequent recognition test. After both exposure phases, participants completed a surreptitious old–new voice recognition task. To further examine whether task awareness is modulated by voice load (number of voice identities introduced in the experiment), we implemented both a simple and a challenging version of the experiment. We found that, irrespective of voice load, implicit learning through participation in a discrimination task, resulted in higher recognition performance than explicit listen-and-memorize training. These findings suggest that highly demanding explicit listen-and-memorize tasks may benefit from incorporating ecologically valid familiarization paradigms, such as voice discrimination. We discuss the implications of our findings in relation to previous empirical research and their relevance for forensic applications.

## Introduction

### Voice learning

Humans learn voices through various forms of exposure and in different contexts. Traditional voice learning experiments typically involve an *explicit* learning phase with a set of voices, either presented in isolation or paired with *e.g.* avatars^2^. In explicit voice-learning tasks, participants are aware that the ultimate goal is to learn voices for subsequent recognition. After the exposure phase, whose form and extent may vary, participants are tested on their ability to recognize the learned voices, either in isolation or among unfamiliar distractor voices^3–5^. Alternatively, participants might be solicited to match learned voices to distinct semantic information they had been associated with (*e.g.*, names, avatars, or numbers^1,2^). From a psychological perspective, *explicit learning* refers to the intentional, conscious, and goal-directed acquisition of knowledge, characterized by deliberate effort, focused attention, and awareness of the learning process and its outcomes^6–8^. Given that explicit tasks dominate experimental research on auditory voice recognition and identification, it may seem somehow surprising that voice learning almost never occurs explicitly in practice. (In line with Kreiman and Sidtis^9^, we use the term recognition to describe the perception that a voice is familiar, without requiring successful speaker naming, which we reserve for identification.) Voices are typically acquired in human speech interaction without conscious awareness that they may serve as identifiers for ’who is talking’ in multi-speaker communication or used in social situations to identify the interaction partner^10^. Here, we refer to any situation in which voices are acquired in the absence of awareness that they serve for subsequent recognition as *implicit* voice learning tasks^6–8^. It is the unintentional acquisition of voice familiarity through extended or repeated exposure^11,12^ or through participation in multi-speaker conversations. Implicit voice learning can also occur during activities such as listening to recorded or broadcast media, without any direct interaction. In this study, we elicited implicit learning by engaging participants in a voice discrimination task requiring same–different judgments, without informing them of a subsequent recognition phase. Different terms are used in the literature to describe contrasting aspects of task awareness (awareness regarding the goal of voice learning), including *implicit* and *incidental* learning, often contrasted with *explicit* or *intentional* learning. For clarity and consistency, we use the terms *implicit* and *explicit* learning throughout this manuscript.

### Factors influencing voice learning in applied contexts

Recognition performance in explicit listen-and-memorize scenarios, such as the Glasgow Voice Memory Test (GVMT)^3^, decreases when more naturalistic stimuli (beyond single vowels) are used, such as read sentences^13^, and can even approach chance level with spontaneous speech^14^. Additionally, as early work by Legge et al.^15^ demonstrated, such experimental designs have been shown to be substantially influenced by voice load (number of voice identities introduced in the experiment). Although a systematic investigation into how the number of to-be-memorized voices affects recognition performance is lacking, previous findings suggest that participants can successfully memorize between four and eight voice identities in the short term^3,4,7,13,15^. By contrast, learning ten or more voices led to a marked decline in recognition performance^15^.

Understanding robust learning of unfamiliar voices is important beyond laboratory settings and has direct relevance for applied contexts such as digital forensics in law enforcement. In these settings, the need to efficiently process speech data has become increasingly urgent as the volume of audio generated in everyday life continues to grow alongside the increased availability of digital biometric traces^16^. In data-intensive investigative contexts involving recordings of variable or poor quality, traditional auditory-acoustic^17^ methods developed for one-to-one voice comparisons are too time-consuming to be used at scale. By contrast, while automatic approaches offer greater efficiency, their performance can decline when applied to poor-quality recordings or short speech samples^18,19^. Consequently, machine-based results require human oversight^20^, and recent work has explored novel approaches that incorporate innate human voice-perception abilities for post-processing^21^. However, it remains largely unclear how listeners can be effectively familiarized with such materials, as explicit listen-and-memorize approaches have been shown to result in low recognition performance under high voice-load (i.e. large numbers of voices in the experiment) conditions^15^.

Interestingly, anecdotal evidence from law enforcement personnel suggests that extended exposure to a voice can lead to robust familiarization—resembling patterns observed in real-world implicit voice learning. For example, in personal communication with the authors, police transcribers have described becoming highly familiar with speakers through prolonged transcription of intercepted conversations. Comparable forms of voice familiarization have also been reported by interpreters when transcribing and translating multi-speaker files. These anecdotal observations contrast with the above-described difficulties associated with explicit listen-and-memorize learning paradigms and suggest a potential benefit of implicit exposure for voice learning.

Beyond task awareness and voice load, previous studies have reported that perceived vocal distinctiveness influences voice recognition performance^22,23^. Distinctiveness has been linked to a voice’s position within an acoustic feature space, such that voices that deviate more strongly from an acoustic prototype are perceived as more distinctive^24^. Accordingly, recent work on voice learning has incorporated acoustic (dis)similarity analyses to vary and control task difficulty^4^. In addition, machine-based similarity metrics derived from Mel-frequency cepstral coefficients (MFCCs) have been shown to correlate with human similarity judgments^25^. Building on this approach, Fröhlich et al.^21^ used MFCC- and F0-based measures for stimulus selection and demonstrated reliable effects on task difficulty in voice discrimination tests.

### Benefits of implicit exposure

Conceptually related research from the field of second language acquisition suggests that implicit training can facilitate the learning of nonnative phonetic contrasts^26^. Another study found that learners acquired stress patterns equally well through explicit and implicit training^27^. Moreover, task-irrelevant implicit exposure has been shown to facilitate the detection of formant transitions^28,29^. Whether these effects extend to other linguistically relevant phonetic categories remains an open question.

Much of our understanding of implicit voice learning in experimental settings stems from research on the *familiar talker effect*–the observation that listeners comprehend speech more effectively when it is produced by voices they have previously been familiarized with^7,30,31,32^. While early studies on the familiar talker effect primarily employed explicit voice learning tasks^32,33^, more recent work has used implicit learning paradigms to investigate how voice familiarization supports speech comprehension^31,34–36^. Several of these studies have also employed voice recognition tests to identify the threshold of familiarity at which the *familiar talker effect* becomes observable. While not primarily aimed at studying implicit voice *learning*, these studies show that voice familiarity can develop implicitly through speech processing, even in experimental settings^31,34,36^.

### Effect of task awareness on voice learning

To date, the few existing studies comparing implicit and explicit voice learning scenarios have reported mixed findings. One possible contributor to this variability is the wide range of experimental designs applied across studies^37^. For example, implicit voice learning has been studied using paradigms that mislead participants about task purpose^38,39^ or designs involving entirely unexpected recognition tests following implicit familiarization^40^. Similarly, explicit learning paradigms differ substantially in their familiarization procedures, for example by requiring participants to learn voices paired with avatars^12^ or to perform simple listen-and-memorize tasks^14,38,40^. Additional differences include the presence or absence of a consolidation phase, as well as variation in the feedback provided.

On the one hand, benefits of explicit voice-learning scenarios have been reported, for example by Armstrong and McKelvie^39^, who found consistently higher recognition performance following explicit listen-and-memorize tasks compared with content-focused implicit exposure. Interestingly, however, they reported above chance performance after both the implicit and explicit exposure phases. Similarly, Humble et al.^37^ investigated task awareness by instructing listeners, prior to hearing each voice, either to remember it or to forget it. Voices designated as to be remembered were recognized significantly more accurately than those designated for forgetting. Importantly, recognition accuracy for voices designated for forgetting remained above chance, suggesting that some degree of voice familiarization occurs even in the absence of explicit learning intentions.

In forensically relevant experimental designs, Cook and Wilding^40^ and, similarly, Perfect et al. ^38^, found comparable voice recognition performance following implicit exposure focused on speech content and explicit listen-and-memorize instructions. These findings were later confirmed by Schäfer ^14^, who likewise reported no advantage of explicit encoding over content-focused implicit exposure for voice recognition.

Interestingly, research on task awareness in voice learning has focused predominantly on implicit situations in which listeners attend to speech content rather than to the voice itself. Other forms of implicit familiarization that involve greater task engagement or a stronger focus on voice features, have received comparatively little empirical attention^12^. Worth noting, however, is early work that investigated task awareness with respect to speaker attributes such as the sex of the speaker. These studies showed that memory for speaker sex was reliably above chance even when listeners attended only to sentence content. Explicit instructions to remember the sex of the speaker provided mixed results^41,43^. Against this backdrop, only one study to date has examined implicit voice learning via participation in a voice discrimination task, without awareness of a subsequent recognition task^12^. This gap is notable given that performance in voice discrimination tasks remains relatively robust even under challenging conditions, including short stimuli, degraded recording quality, and mismatches in speaking style^21,43^. Moreover, because voices in natural communication are rarely encountered in isolation, voice learning typically unfolds in the presence of multiple, competing talkers. Together, these considerations position discrimination-based implicit learning as a promising yet underexplored approach.

The only other study that examined implicit voice learning through speaker discrimination is that of Lee and Perrachione^12^. Their study comprised three tasks that differed in the level of task awareness. In the explicit learning condition, referred to as “talker matching”, participants learned to match voices to corresponding avatars, a task that went beyond a simple listen-and-memorize paradigm incorporating feedback. In the “talker 1-back” task, participants judged whether a voice heard in one trial matched a voice presented in the immediately preceding trial. This task resembled a voice discrimination task. Although this task required attention to vocal characteristics, it did not explicitly require participants to learn or memorize individual talker identities and can therefore be considered implicit in the sense that participants had no expectation of learning voices for subsequent recognition^12^. Finally, the “verbal 1-back” task required participants to judge whether the spoken content, rather than the voice, matched that of the preceding trial. This task is comparable to implicit learning paradigms used in previous studies of task awareness that focus on speech content rather than speaker identity.

Participants were randomly assigned to one of three exposure tasks (between-subjects design) and completed it twice: once in a *familiar* condition, in which the voices presented during learning and test were the same, and once in an *unfamiliar* condition, in which the voices differed across phases. Prior familiarization with voices, even implicitly, without explicit instruction to learn them, was associated with higher recognition performance^12^. The effect was strongest and statistically significant in the explicit voice-learning task, but weakest and non-significant in the voice discrimination task (“talker 1-back”), suggesting that prior familiarization through discrimination conferred no significant benefit for subsequent voice learning. This latter finding was unexpected, as voice discrimination was predicted to at least partially mirror everyday listening conditions in which multiple voices are encountered simultaneously. The authors of this study, Lee and Perrachione^12^ suggested that the limited benefit may stem from low task difficulty, which might not have sufficiently engaged participants. Another possible explanation for the limited learning benefits is that the use of spoken digits as stimuli may have provided less speaker-specific information than sentences, thereby constraining learning.

Taken together, several key questions remain open. Most notably, discrimination-based implicit learning has yet to be compared to explicit listen-and-memorize paradigms, which have been employed in the majority of studies examining the effects of task awareness. Additionally, it remains to be tested whether potential benefits of implicit learning through discrimination might emerge under more naturalistic conditions that require higher task engagement, such as degraded recording quality or increased voice similarity. Finally, all prior studies have examined task awareness using between-subject designs, leaving open the possibility that observed effects are confounded by individual differences in voice-learning ability.

### The current study

Given these open questions, the present study is the first to directly compare voice recognition performance following an implicit voice-discrimination task, in which listeners were unaware of a subsequent memory test, with performance following an explicit listen-and-memorize task modeled after the GVMT^3^. All participants completed both implicit and explicit learning conditions, with the order of the two procedures counterbalanced across participants. After both exposure phases, they completed an old/new recognition task. This within-subjects design minimized the influence of individual differences in voice learning ability and ensured that performance comparisons were not confounded by between-group variability.

As listen-and-memorize tasks have been shown to be influenced by voice load, participants completed either a simple (four to-be-learned-voices) or a challenging version (ten to-be-learned-voices) of the experiment. To ensure comparable task complexity with respect to voice distinctiveness, stimulus selection was conducted using an automatic speaker-recognition-based method^21^.

Several considerations motivate our hypotheses regarding the expected results. First, previous work has shown that explicit listen-and-memorize tasks yield recognition performance comparable to that observed following content-focused implicit exposure^14,38,40^. Because voice discrimination requires greater task engagement than content-focused implicit exposure, it may more effectively support voice learning even in the absence of explicit instructions to memorize voices for later recognition. Second, voices in natural communication are rarely encountered in isolation but are typically learned in dialogic or multi-speaker contexts. We therefore hypothesize that implicit learning through voice discrimination will lead to superior recognition performance compared with explicit listen-and-memorize paradigms. Although the findings by Lee and Perrachione^12^ contradict this expectation, their results may be attributable to low engagement in the discrimination task and to its comparison with an extensive, explicitly instructed voice-to-avatar matching paradigm that included feedback. In contrast, the present study focuses on simpler listen-and-memorize learning scenarios, as these allow the investigation of faster and more ecologically valid familiarization, despite potentially reduced robustness.

Furthermore, we expect recognition performance to decrease as voice load increases, resulting in lower accuracy in the more challenging version of the experiment. Finally, we anticipate that the advantage of implicit learning will be particularly pronounced under challenging conditions, where reduced task demands may support more effective learning.

## Methods

### Participants

The initial sample comprised 133 police cadets from the Zurich Cantonal Police. One participant was excluded from the analyses due to technical issues. Of the remaining 132 participants, 63.64% reported Zurich German as their first language, 34.85% reported other Swiss German dialects, and two participants reported Standard German acquired in Germany as their first language.

In the simple version of the experiment, 81 participants (46 self-assigned men and 35 women) were tested, while in the challenging version of the experiment, 51 participants (30 self-assigned men and 21 women) were tested. Participants were on average $27.6 \pm 3.9$ years old (range: 21–37, median: 27).

The study was approved by the Ethics Committee of the Faculty of Arts and Social Sciences at the University of Zurich and in accordance with the Declaration of Helsinki. All participants provided informed consent before beginning the experiment.

### Stimuli

The speech materials were taken from the original version of the Temporal Voice Idiosyncrasy (TEVOID) corpus, which comprises recordings of read and spontaneous sentences produced by native speakers of Zurich German, the Alemannic dialect spoken in the city of Zurich and most of the surrounding canton^44^. For the current study, only read speech materials were used. Importantly, although these were read sentences, they were derived from naturally produced, originally spoken phrases. Consequently, they are assumed to retain some characteristics of spontaneous speech and to exhibit greater intra-speaker variability than read sentences. The speakers were on average $30.3 \pm 6.6$ years old (range: 18–32). All recordings were made in studio quality and digitized at a sampling rate of 44.1 kHz with a 16 kbit/s bitrate.

A novel stimulus selection method (see description below) was applied to select speakers from the TEVOID corpus, resulting in a subset of four male and four female speakers for the simple version of the experiment, and ten male and ten female speakers for the challenging version. Stimulus pre-processing comprised the removal of non-speech sections before and after the utterances, segmentation into 1.2-second snippets extracted from the midpoint of each recording, and amplitude normalization to 65 dB root mean square (RMS). In addition, amplitude smoothing was applied using Praat^45^. Finally, the stimuli were down-sampled to 8 kHz to correspond to a frequently observed setting in forensic casework (e.g., telephone-transmitted speech).

**Table 1:** Assigning voice identities to the different tasks from a ranked list of similarities.

#	Learned voices (old)	Impostor voices (new)	Task awareness	Similarity
1	speaker_a.mp3	speaker_b.mp3	implicit	decreasing
2	speaker_c.mp3	speaker_d.mp3	explicit	$\downarrow$
3	speaker_e.mp3	speaker_f.mp3	implicit	
4	speaker_g.mp3	speaker_h.mp3	explicit	
5	....	.....	...	

### Stimulus selection: controlling acoustic voice distinctiveness across tasks

Previous work has shown that certain voices are inherently more distinctive and memorable than others^22^. In our study, we used different sets of voices for the implicit and explicit learning tasks. To minimize the possibility that one set of voices was systematically easier to memorize and recognize than the other, we used an automated method to assess and control acoustic (dis)similarity between voices. We combined similarity scores obtained from an automatic speaker recognition software (ASR) with independently extracted measures of fundamental frequency (F0) and based speaker selection on both metrics. This approach was similar to the one previously described by Fröhlich et al.^21^.

As ASR-software, we used the Emphasized Channel Attention, Propagation and Aggregation in TDNN Based Speaker Verification (ECAPA-TDNN) deep learning model available from Speechbrain^46^ (version 0.5.10, release 05-03-21,

https://github.com/speechbrain/speechbrain/releases/tag/0.5.10) in Python (version 3.8,

https://www.python.org/downloads/release/python-380/) due to its ability to perform well on short stimuli^47,48^.

We started by loading all 256 sentences for each of the TEVOID speakers using the Audio Normalizer module. Subsequently, we generated embeddings utilizing the EncoderClassifier classes from Speechbrain. We then calculated pairwise cosine similarities between the embedding vectors to obtain a full similarity matrix for all sentence files. From this, a consolidated matrix that encompassed all speakers along with their averaged similarities to all other speakers was computed. During the extraction of MFCC features, which are used to train the ECAPA-TDNN, a significant portion of F0 information is disregarded. Nonetheless, previous studies have established that mean pitch can significantly affect listeners’ perceptions of voice (dis)similarity^49,50^. We therefore further measured and averaged the fundamental frequencies (F0) for all speakers and calculated mean delta F0 values for all potential speaker pairs within one gender. These delta F0 values were added to the existing similarity matrix as additional columns (same number as ASR cosine similarities). The result was a table that included all averaged cosine similarities and delta F0 values per speaker pair (separated by gender). No likelihood ratios were calculated, as the system was solely used on a score basis.

Based on cosine similarities from ASR systems and delta F0 values, we computed pairwise Euclidean distances between all speakers (all features weighted equally). Voice selection for the tasks was therefore based on speaker identity, using the average similarity across all files per speaker. Speaker pairs were then ranked by similarity, with the most similar pairs appearing at the top of the list. Each to-be-learned voice, used either in the discrimination or memorization task, was paired with one impostor voice, which appeared only in the test phase, to balance task complexity. The “train” set included voices participants were instructed to memorize and later recognize as “old”, while the “test” set consisted of impostor voices that had to be correctly identified as “new”. For each speaker pair, we randomly selected one of the 256 available sentences from the target speaker (learned voice) for use in the learning phase, and one sentence from the paired impostor speaker for use in the test phase (see Table 1). Each file and sentence snippet was only used once.

By assigning these pairs alternately to the implicit and explicit voice learning tasks, we controlled task complexity and ensured both modalities were comparable. The stimulus selection method is visualized in more detail in Table 1. In total, the simple version included four to-be-learned voices in each exposure phase (implicit and explicit), whereas the challenging version included ten voices in each exposure phase. Importantly, the similarity range from which stimuli could be selected was constrained by the composition of the original corpus.

### Procedures

The experiment consisted of two exposure phases and two test phases. Each participant began with either an implicit or explicit exposure phase, followed by a corresponding voice recognition test. The exposure condition was counterbalanced across phases, such that participants experienced both types of exposure in alternating order (see Table 2). The experiment was implemented using the Gorilla Experiment Builder^51^. Participants completed the experiment on-site with all participants wearing the same headphones (Sennheiser HD25, 70 Ohm). Stimulus presentation was not automated; each stimulus was initiated manually by pressing *PLAY*. After playback ended, the program advanced automatically to the subsequent trial. Consequently, the procedure was self-paced, allowing participants to take brief pauses before initiating the next stimulus.

**Table 2:** Experimental design for the four participant groups, with task order presented from left to right; e.g I $\rightarrow$ E (implicit first, explicit second). Emphasis indicates learning condition: bold : implicit learning through voice discrimination; italic: explicit learning through memorization.

Version	Order	Exposure phase 1	Test phase 1	Exposure phase 2	Test phase 2
Simple	I $\rightarrow$ E	**Voice discrimination**	Voice recognition	*Voice memorization*	Voice recognition
	E $\rightarrow$ I	*Voice memorization*		**Voice discrimination**	
Challenging	I $\rightarrow$ E	**Voice discrimination**	Voice recognition	*Voice memorization*	Voice recognition
	E $\rightarrow$ I	*Voice memorization*		**Voice discrimination**	

#### Manipulating task awareness: implicit vs. explicit

The *implicit* voice exposure phase encompassed a binary same-different voice discrimination task similar to the Bangor Voice Matching Test (BVMT)^52^. Stimuli were 1.2 seconds long; an interstimulus interval of 1 s was implemented and sentence snippets were used. Participants heard each target voice a total of eight times during exposure—four times in same-speaker trials and four times in different-speaker trials—with a different sentence snippet presented on each trial. This resulted in exposure to 12 distinct sentence snippets per voice identity. In the *explicit* exposure phase, participants completed a prompted voice memorization task in which, for each voice identity, 12 sentence snippets (each 1.2 s long, with 0.5-s interstimulus pauses) were presented. Between each to-be-memorized voice, a 3-s interval was presented using a fixation cross. Importantly, the total exposure time per voice identity was identical across the implicit and explicit conditions.

After each of the two voice exposure phases, the participants completed a subsequent old-new voice recognition task. During each recognition trial participants were required to indicate whether the voice (1.2 s long stimulus) was one they had heard previously (“old”, known from the exposure phase) or if it represented a “new” voice identity. Each voice identity was tested in three trials. Importantly, the sentence snippets in the recognition trials differed from those used in the learning phase. Alongside the previously introduced voice identities, impostor voices were played (each similar to one of the learned voice identities). No feedback was provided throughout the experiment. Since all participants completed both the implicit and explicit voice-learning phases, this part of the experiment was conducted using a within-subjects design.

#### Manipulating voice load: simple (4 voices) vs. challenging (10 voices)

To explore whether differences in task awareness are affected by voice load, we implemented two experiment versions. In the simple version of our experiment, participants were exposed to two sets of four voices, consistent with previous work using eight target identities in total^3,14^. This resulted in a total of 30 discrimination trials and 24 recognition trials. Explicit exposure was presented in a single block, as described above. In the challenging version of the experiment, two sets of ten distinct voice identities were included; consequently, to ensure the same exposure duration per voice identity across the simple and challenging versions, the number of discrimination trials was doubled from 30 to 60. Likewise, to ensure that each voice was tested three times, the number of recognition trials was increased to 60. Explicit exposure was likewise presented in a single block. This manipulation allowed us to explore the limits of implicit voice learning under conditions that more closely approximate real-world variability and memory demands. Importantly, the voice exposure duration was identical for each voice identity in both the simple and challenging versions of the experiment. Only the *number* of introduced voices differed. Further details on differences between the simple and challenging experiment versions can be found in the Supplementary Information Table S1. Each participant completed either the simple or the challenging version of the experiment; therefore, this part of the study was conducted using a between-subjects design. The experiment duration for the challenging version had a mean of 33.37 min (SD = 2.43), whereas the simple version had a mean duration of 17.20 min (SD = 1.67).

### Analyses

All statistical analyses were performed in Python (version 3.8, https://www.python.org/downloads/release/python-380/). Descriptive and inferential statistics were conducted using the scipy.stats module (version 1.10.1). For linear mixed-effects models, we used the pymer4 package^53^(version 0.8.4), a Python interface to the lme4^54^ and lmerTest^55^ packages in R (version 4.1.3, https://cran.r-project.org/bin/windows/base/old/4.1.3/). The mixed-effects models were fitted using maximum likelihood estimation, with *p*-values computed via Satterthwaite’s approximation as implemented in lmerTest. Non-parametric tests were used when assumptions of normality were violated, including the Shapiro–Wilk test for assessing normality^56^ and Spearman’s rank correlation coefficient $\rho$^57^ for correlation analyses. Group-level comparisons were conducted using Welch’s unequal variances *t*-test^58^, and *p*-values were adjusted for multiple comparisons using the Bonferroni correction^59,60^. Plots were generated using seaborn (version 0.13.2) and matplotlib (version 3.7.2). Sensitivity ($d'$); a measure of the ability to discriminate between target and non-target voices was calculated following Stanislaw and Todorov ^61^. A hit was defined as trials in which an *old* (previously heard) voice was correctly identified as “old”, and false alarms were trials in which a *new* (previously unheard) voice was incorrectly judged as “old”. Individual $d'$ scores were calculated as the difference between the z-transformed hit rate and false alarm rate:

$$\begin{aligned} d' = Z(\text {hit rate}) - Z(\text {false alarm rate}) \end{aligned}$$

To avoid infinite values resulting from hit or false alarm rates of 0 or 1, we applied a standard correction^62^.

#### Learning phase: assessing difficulty of voice discrimination task

Previous findings suggested that low task difficulty, and consequently low participant engagement, can influence voice learning^12^. Therefore, the level of difficulty of the implicit voice learning task based on voice discrimination was assessed using descriptive statistics of $d'$ (mean, median, standard deviation), and by examining distribution skewness and potential ceiling effects with a chi-square test. A soft ceiling threshold set at the 90th percentile of the $d'$ distribution for each condition was used in the chi-square analysis.

#### Test phase: assessing effect of task awareness on voice recognition performance

For the descriptive analyses, we computed the mean, median, and standard deviation. We then examined correlations between exposure performance (voice discrimination in the implicit exposure phase) and test performance (recognition test after discrimination). Normality of the data was evaluated using the Shapiro–Wilk test^56^. Given that this assumption was not met, we conducted non-parametric correlation analyses using Spearman’s rank correlation coefficient^57^. To investigate the effects of task awareness (implicit vs. explicit), voice load (simple vs. challenging) and task order (exposure phase 1 vs. 2) on subsequent voice recognition performance (measured as $d'$) we fitted a linear mixed-effects model using a Gaussian distribution. We fitted a single model across both experiment versions (varying in voice load) to account for a possible interaction between exposure type (implicit vs. explicit), voice load (simple vs. challenging) and task order (which task is in exposure phase 1 and which in phase 2). See model formula (Eq. 1) including interaction terms (see Supplementary Information for results of interaction analysis) and the final formula (Eq. 2):

1 $$\begin{aligned} \texttt {dprime} \sim \ \texttt {task awareness} * \texttt {voice load} * \texttt {task order} + (1|\texttt {participant}) \end{aligned}$$

2 $$\begin{aligned} \texttt {dprime} \sim \ \texttt {task awareness} + \texttt {voice load} + \texttt {task order} + (1|\texttt {participant}) \end{aligned}$$

#### Further analyses

#### Voice recognizability across varying voice load

To better understand how voice load influences voice recognizability, we compared recognition accuracy (measured as percent correct, PC) between the simple and challenging versions of the experiment. This analysis focused on the subset of voice identities shared across both conditions. For each voice identity, PC scores were computed separately for the simple and challenging conditions.

#### Recognizability of learned vs. impostor voices

To ensure comparable distinctiveness between learned and impostor voices across both learning and test phases (implicit and explicit), we implemented a novel stimulus selection method based on automatically computed Euclidean distance measures using MFCC and F0 features (see section “stimulus selection” for details). Given the novelty of this approach, we conducted an additional analysis to assess whether the automatically assigned impostor voices (“new”) were as difficult to recognize as the learned voices (“old”). To do so, we computed PC scores at the speaker level and compared them across conditions. “New” vs. “old” speaker were considered significantly more or less recognizable if the corrected *p*-value was below the significance threshold of $\alpha =.05$.

## Results

### Learning phase

In the discrimination task, the mean $d'$ for the challenging experiment version *(n = 51, trials = 60)* was 2.02 (*SD* = 0.45; median = 2.07). The simple version *(n = 81, trials = 30)* showed a non-significantly lower mean of 1.96 (*SD* = 0.41; median = 1.95). An independent-samples *t*-test confirmed that this difference was not statistically significant ($t(130) = -0.86,\, p =.393$.) With regard to the investigation into potential ceiling effects; no significant skewness was observed in either the simple or challenging version, and no participant reached the absolute maximum score. Further chi-square analysis based on $d'$ values revealed that, in the challenging version, 6 of 51 participants (11.8%) scored at or above the soft ceiling threshold of $d'$ = 2.58, which did not differ from the expected count (5.34), $\chi ^2$(1) = 0.09, *p* = .764. Similarly, in the simple version, 9 of 81 participants (11.1%) scored at or above the soft ceiling threshold of $d'$ = 2.43, which did not differ from the expected count (10.48), $\chi ^2$(1) = 0.24, *p* = .623. These results therefore provide no evidence of ceiling effects in either experiment version. See Fig. S1 in the Supplementary Information for $d'$ distributions of the discrimination tasks.

### Test phase

**Fig. 1:**
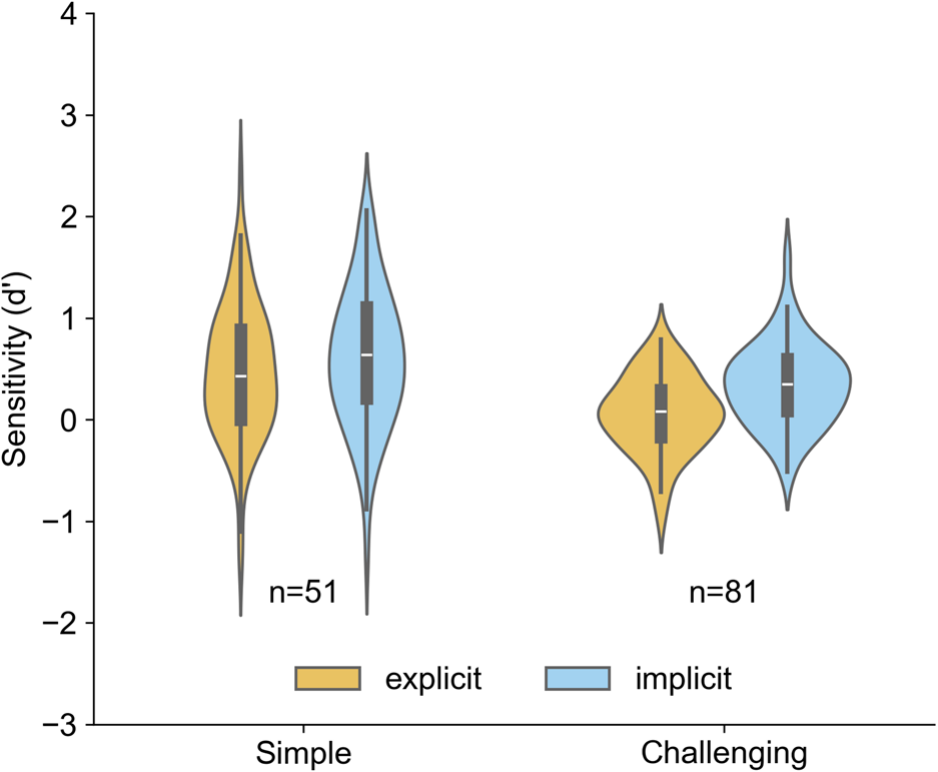
Speaker recognition results (measured as $d'$) for simple and challenging experiment versions and both exposure types.

See Fig. 1 and Table 3 for an overview of the $d'$ results for the recognition tasks after both (implicit and explicit) exposure phases. Both the violin plots in Fig. 1 and the $d'$ results in Table 3 suggest that the observed difference in $d'$ means is more pronounced in the challenging version ($\Delta M$ = 0.285) than in the simple version ($\Delta M$ = 0.12).

**Table 3:** Descriptive statistics of sensitivity ($d'$) by condition.

Condition	Mean $d'$	Median $d'$	Std $d'$
Simple Explicit (n=81)	0.472	0.431	0.653
Simple Implicit (n=81)	0.592	0.641	0.679
Challenging Explicit (n=51)	0.056	0.084	0.379
Challenging Implicit (n=51)	0.341	0.347	0.408

The results of the regression analysis can be found in Table 4. We found main effects of task awareness (implicit vs. explicit) on $d'$ (estimate = 0.186, 95% CI [0.051, 0.321], *p* = .008). Implicit voice exposure via the discrimination task was associated with significantly higher $d'$ values, independent of voice load (simple vs. challenging). Voice load also showed a strong positive effect (estimate = 0.333, 95% CI [0.186, 0.480], *p* < .001), indicating that the challenging version yielded significantly lower recognition performance compared to the simple version. By contrast, task order did not significantly affect performance (estimate = –0.073, *p* = .289). No significant interactions were found between task awareness, voice load, and order. A supplementary between-subjects analysis, considering only Task 1 from all participant groups, is provided in the Supplementary Information.

**Table 4:** Regression model output showing estimates, 95% confidence intervals (CI), standard errors (SE), degrees of freedom (df), t-statistics (*t*), p-values (*p*), and significance levels (Sig).

Term	Estimate	2.5% CI	97.5% CI	SE	df	t	p	Sig
Task awareness [explicit]	0.186	0.051	0.321	0.069	130.0	2.696	0.008	**
voice load [challenging]	0.333	0.186	0.480	0.075	130.0	4.440	< 0.001	***
Order [phase 1]	$-0.073$	$-0.209$	0.062	0.069	130.0	$-1.065$	0.289	

**Fig. 2:**
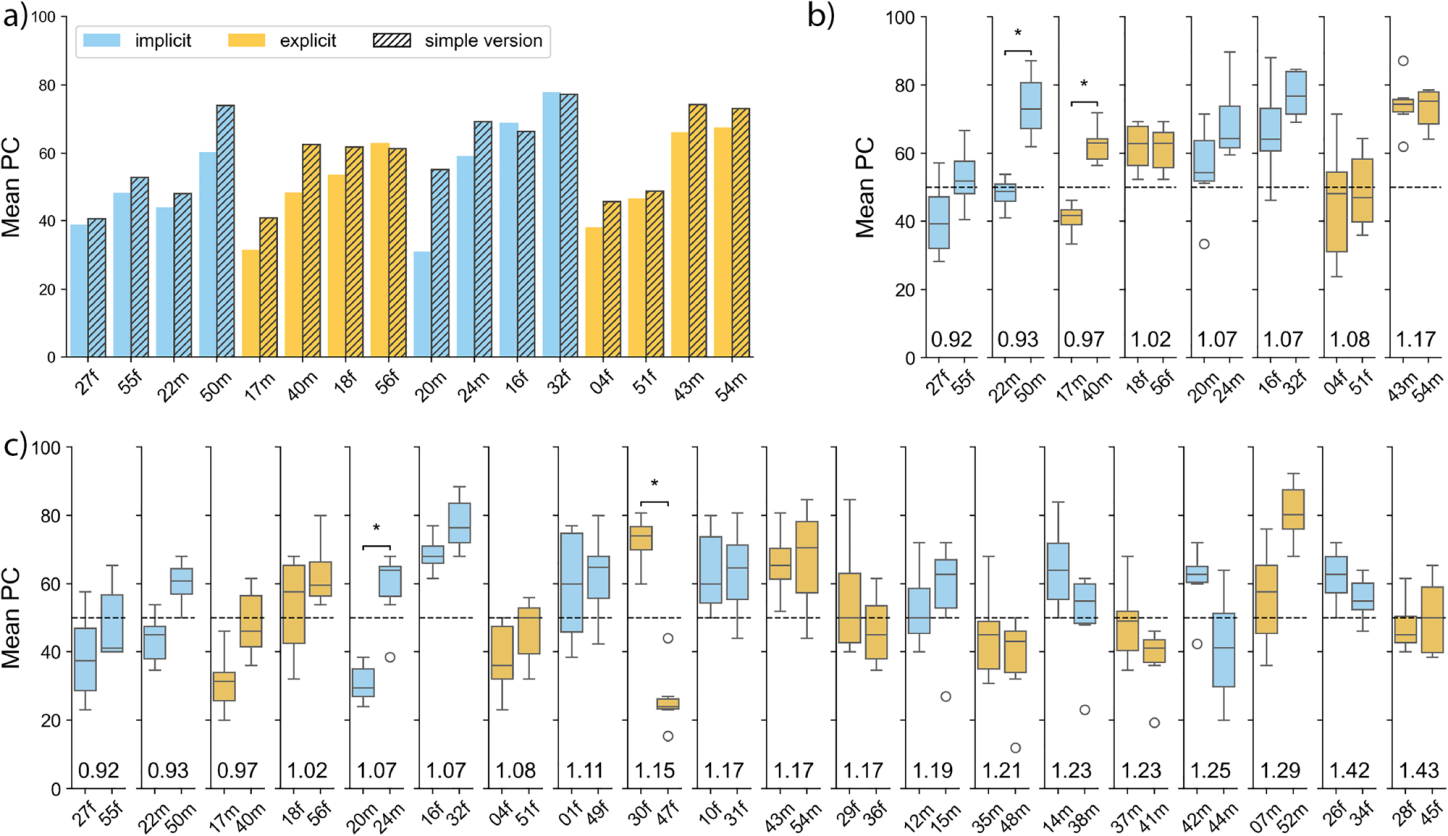
Analysis of voice recognizability per voice identity measured as mean PC across all participants. (**a**) Simple and challenging experiment versions; (**b,c**) Comparisons between learned (left, e.g. “27f”) and impostor voices (right, e.g. “55f”) in the simple and challenging versions, respectively. Speaker pairs are ranked by increasing Euclidean distances (number above each pair) from left to right. Significance assessed at $\alpha = 0.05$; Asterisks above significance brackets indicate Bonferroni-corrected p-values. Dashed line indicating chance level (50%).

### Correlation between learning and test phase

No significant correlation was found between the participants’ performance in the discrimination task (implicit exposure) and the subsequent recognition task involving the same voices for the simple ($\rho$ = –.168, *p* = .133) nor the challenging ($\rho$ = .154, *p* = .281) version of the experiment.

### Further results

#### Recognizability across simple and challenging version

We compared mean PC scores for the subset of speakers appearing in both the simple and challenging experiment versions, see Fig. 2a. No significant differences in recognizability were found for any voice identity.

#### Voice recognizability of learned vs. impostor voices

Differences in voice recognizability between learned voices (via implicit or explicit exposure) and their impostor counterparts were quantified using PC scores at the speaker level. In the simple experiment version, only 2 out of 8 speaker pairs showed statistically significant differences in recognizability between learned and impostor voices. Similarly, in the challenging version, only 2 out of 16 pairs showed significant differences. These results indicate that, for most voice identities, impostor voices were recognized as old/new with similar difficulty as the learned voices (see Fig. 2b,c).

## Discussion

This study examined whether a voice-discrimination task, implicit with respect to voice learning, yields superior voice recognition performance compared to an explicit listen-and-memorize task. All participants completed two exposure phases, each of which was followed by an old–new recognition test. Furthermore, to assess the effect of varying voice load, two versions of the experiment were implemented: a simple and a more challenging version. The results yield three main insights.

First, consistent with our primary hypothesis, the voice-discrimination task (implicit with regard to voice learning) produced higher recognition performance than the explicit listen-and-memorize task. This novel finding extends earlier studies showing *comparable* recognition performance following explicit listen-and-memorize training and content-focused implicit exposure^14,38,40^. Our novel results show that, when these listen-and-memorize tasks are contrasted with a voice-discrimination task that is implicit with respect to voice learning and places a stronger focus on voice processing than content-based implicit tasks, the benefits of implicit exposure emerge. These findings raise important questions about the field’s predominant reliance on explicit listen-and-memorize paradigms in voice-learning research, particularly in scenarios involving high voice load^15^.

Our findings stand in contrast to those of the only other study investigating voice discrimination as a form of implicit voice learning, which reported no significant advantage of implicit learning through discrimination^12^. The divergence between results may be attributable to differences in experimental design during both the implicit and explicit exposure phases. The authors, Lee and Perrachione^12^, discussed that the limited benefits of implicit learning through their voice discrimination task might stem from the task being too easy, thereby reducing participant engagement. In comparison, the observed mean of our $d'$ scores indicates that our task was considerably more challenging. This increased difficulty may have facilitated learning, thereby making the advantage of implicit over explicit learning possible. In addition, differences between our *explicit* learning task and that of Lee and Perrachione^12^ may have further contributed to the contrasting results. Our explicit learning phase followed the Glasgow Voice Memory Test (GVMT^3^), which involved repeated listening for memorization and resulted in notably low recognition performance. Results of comparable magnitude have been reported in other studies using the GVMT paradigm^14,63^, suggesting that this approach may be less effective for voice learning with naturalistic stimuli. In contrast, the explicit learning task in the study by Lee and Perrachione^12^ incorporated more thorough voice learning through voice-to-avatar matching with feedback, yielding high identification accuracy and reflecting more robust voice representations. Therefore, we hypothesize that the observed advantage of implicit learning may, at least in part, reflect limitations in the effectiveness of the explicit learning phase, a consideration that is important for interpreting differences in learning outcomes.

A potential explanation for the lower recognition scores following the explicit listen-and-memorize tasks is the high cognitive load imposed by this learning scenario. Cognitive load theory^64,65^ proposes that working memory has a limited capacity for processing information. According to this theory, cognitive demands arise not only from the complexity of the material itself (intrinsic load) but also from the way information is presented or the nature of the task demands (extraneous load). In our study, instructing participants to deliberately memorize voices may have imposed such extraneous cognitive load–redirecting attention away from the perceptual processing of the voices and overburdening working memory. In contrast, exposing participants to a voice-discrimination task, without instructions to memorize the voices and with task demands more closely aligned with perceptual processing, may have reduced cognitive load. This assumption is motivated by the generally high performance reported for voice-discrimination tasks, suggesting a relatively low cognitive load during task execution (see, for example,^21^, who used the same stimuli).Secondly, we hypothesized that the challenging version of the experiment would result in lower overall recognition performance compared to the simple version, which was confirmed. This finding aligns with previous work showing that increasing voice load can impair recognition accuracy^15^.

Finally, we hypothesized that the advantage of implicit exposure would be particularly pronounced in the more challenging version of the experiment, as explicit listen-and-memorize tasks are known to lead to marked performance declines under high voice load^15^. In such demanding situations, implicit tasks may be especially beneficial by reducing cognitive demands and thereby supporting more efficient learning. Although no significant interaction between experiment version and task awareness was observed, descriptive results indicate that the difference in recognition performance between implicit and explicit learning conditions was larger in the challenging version (Fig. 1, Table 3); this pattern should be interpreted with caution.

### Implications for studies on voice learning

Numerous studies, including our explicit learning task, have adopted voice-learning paradigms modeled after the GVMT^3^, which feature an explicit learning phase in which participants memorize voices through listening, followed by an old–new recognition task^13,14,63,66^. Taken together, our findings call into question whether instructing participants to listen to and memorize voices for later recognition constitutes the most effective learning strategy when naturalistic stimuli are used. Instead, they suggest that voice discrimination, implicit with respect to voice learning, may offer a more efficient and ecologically valid alternative under such conditions.

However, because the mean $d'$ values in the present study were considerably lower than those reported for the original GVMT, our observations may primarily generalize to conditions employing more naturalistic stimuli beyond single vowels. Consistent with this interpretation, previous studies have reported declines in recognition performance following listen-and-memorize tasks for read sentences, and in some cases performance has even approached chance level when spontaneous speech was used^13,14,63^. To determine whether the observed advantage of implicit exposure extends to the original GVMT stimuli, re-running the GVMT within an implicit voice-discrimination framework would provide a critical test of the generalizability of the present findings across different stimulus types.

### Implications for forensic settings

One motivation for the present study was to gain deeper insight into how listeners can be optimally familiarized with voices under conditions involving high voice loads. Such scenarios frequently arise in law enforcement contexts, where large sets of case-relevant recordings are screened for a specific target voice, often under degraded recording conditions^21^. In these data-intensive investigative settings, traditional one-to-one voice comparison methods^17^ are poorly suited to operational demands due to their limited scalability, while automatic approaches can be affected by recording quality and speech duration^18,19^. Together, these challenges have contributed to a growing interest in human voice-perception as a complementary element within investigatory workflows^21^. However, there remains limited understanding of how listeners should best be familiarized with voices to support robust performance in such contexts. Our novel findings tentatively suggest that voice discrimination and voice learning are related processes, such that implicit exposure to voices during discrimination tasks can foster speaker familiarity and support subsequent recognition performance. Rather than familiarizing listeners through effortful listen-and-memorize tasks, exposing them to discrimination-based tasks could be used as an implicit means of voice learning.

### Limitations and future directions

Notwithstanding our motivations, our methodological choices were inevitably associated with certain limitations. Firstly, one key concern is that task *awareness* and task *engagement* may have been confounding factors: While in the explicit voice memorization task, participants “passively” listened to recordings, in the discrimination task, they were required to “actively” decide and respond (“same” or “different”) in order to proceed. This discrepancy in cognitive involvement could have contributed to the observed differences in performance. Importantly, differences in task engagement have also been manipulated in prior studies examining task awareness^12,41,42^. More broadly, a lack of clear definitions of task awareness and substantial variability in experimental designs have previously been criticized. For example, Humble et al.^37^ discussed the wide range of implicit experimental paradigms used in studies of task awareness. Accordingly, to ensure that the present findings are not primarily driven by differences in task engagement, future studies should aim to equate engagement levels across implicit and explicit learning tasks.

With regard to differences between our implicit and explicit tasks beyond task awareness, it is also important to note that the stimulus presentation formats differed between the two conditions. In the implicit voice discrimination task, stimuli were presented pairwise across multiple voices, rather than in single-speaker blocks as in the explicit listen-and-memorize task. It is currently unknown to what extent these differences may have influenced learning outcomes and therefore constitute an additional limitation of the present study.

Secondly, performance was overall poor in both experiment versions (simple and challenging) across both recognition tasks (following implicit and explicit exposure). This observation was especially pronounced after the explicit memorization phases of the challenging experiment version. These results therefore raise questions about the effectiveness of our explicit learning task. Future work should thus investigate whether the benefit of implicit over explicit learning persists with intensified training, for example by implementing a consolidation or training phase. However, because the benefits of implicit exposure remained consistent across both versions of the experiment, even in the simpler version in which performance after the explicit learning task was higher, insufficient learning during the explicit exposure phase cannot fully explain the observed benefit of implicit learning in voice discrimination.

Thirdly, although our voice-discrimination task was implicit in the sense that participants were not instructed to learn voices for later recognition, it nevertheless required attention to voice-specific information. It is therefore arguably the case that our voice discrimination can be considered implicit but it is rather a matter of definition. We argue that it qualifies as an implicit task with respect to voice learning, as participants were unaware of the subsequent recognition test. A similar argument was made by Lee and Perrachione^12^. However, to further investigate the effect of task awareness on voice discrimination, future work might entail an experimental design in which participants are informed about a subsequent recognition task prior to starting the voice discrimination task. Related, one might argue that voice discrimination ceased to be implicit when it was performed as the second task by half of the participants. However, participants had no reason to anticipate an additional recognition task based on the discrimination phase. Importantly, task order was not a significant predictor of performance, and an additional analysis restricted to the first exposure phase only, thus implementing a between-subjects design, revealed the same overall pattern of results, further strengthening this interpretation.

Finally, because discrimination performance was less affected by voice load, as reflected in comparable performance across simple and challenging discrimination conditions, and because recognition performance was consistently higher following discrimination-based learning, these findings suggest that voice discrimination may provide a less effortful way of learning voices and/or a way by which the presentation of contrastive voice acoustics lead to better acquisition performance.

### Conclusion

This study investigated how task awareness influences short-term voice learning by comparing explicit listen-and-memorize learning with implicit exposure through voice discrimination. Across two versions of the experiment that varied in voice load, we found that participants consistently demonstrated higher voice recognition performance following implicit exposure through participation in a voice discrimination task. These results suggest that, familiarization through attending a voice discrimination task may be more effective than explicit listen-and-memorize tasks, particularly when cognitive demands are high, e.g., due to high voice load.

Given the limited research on implicit voice learning, these results offer valuable insights into the still incomplete understanding of unfamiliar voice processing. Our findings have both theoretical and practical implications. Theoretically, they contribute to a growing body of evidence emphasizing the importance of ecological validity in voice learning research. Practically, they point toward more efficient and naturalistic approaches for voice learning–especially in applied settings such as forensics, where rapid and accurate voice recognition is critical.

While further research is needed to confirm these effects and explore the underlying mechanisms, our study highlights the potential of voice discrimination tasks as a powerful tool for facilitating implicit voice learning. We encourage future work to continue investigating learning conditions that better reflect the real-world contexts in which listeners encounter and remember unfamiliar voices.

## Supplementary Information

Supplementary Information.

## Data Availability

For access to the Tevoid Corpus contact Volker Dellwo, volker.dellwo@uzh.ch. Behavioral data and analyses code are publicly available in the study’s Open Science Framework repository: https://doi.org/10.17605/OSF.IO/GWTH8.
