# Supplementary material for: Implicit voice learning through discrimination outperforms explicit listen-and-memorize tasks
Source: Sci Rep. 2026 Mar 14;16:13498. doi: 10.1038/s41598-026-41541-z (PMC13111622; doi:10.1038/s41598-026-41541-z)
Supplement: Supplementary file 1 — Supplementary Information. [file 41598_2026_41541_MOESM1_ESM.pdf]

Supplementary Information:

Implicit Voice Learning Through Discrimination Outperforms Explicit Listen-and-Memorize Tasks

Andrea Fröhlich[1,2,4*], Meike Ramon[4,5], Peter French[3,6], and Volker Dellwo[1,3]

[1]Phonetics & Speech Sciences, Department of Computational Linguistics, University of Zurich, Zurich, Switzerland
[2]Speech & Audio Group, Zurich Forensic Science Institute, Zurich, Switzerland
[3]Centre for Forensic Phonetics and Acoustics, Department of Computational Linguistics, University of Zurich, Zurich, Switzerland
[4]Applied Face Cognition Lab, Institute of Applied Data Science and Finance, Bern University of Applied Sciences, Switzerland
[5]AIR—Association for Independent Research, Zurich, Switzerland
[6]JP French International, York, GB
[*]andrea.froehlich@uzh.ch

Table of contents

List of Figures

S1 Voice discrimination performance (measured as d') for the simple a) and b) challenging experiment version . . S2

List of Tables

S1 Specifications on task and experiment version. S2
S2 Pymer interaction estimates S2

Table S1. Specifications on task and experiment version.

Version	# Voices	# Trials	Stimulus per voice identity
		**Implicit exposure**	
CHALLENGING	10	60: 40 SS / 20 DS<br>4 trials each per voice identity	2 x 1.2 s<br>interstim. interval: 1 s
SIMPLE	4	30: 16 SS / 14 DS<br>4 trials each per voice identity	
		**Explicit exposure**	
CHALLENGING	10	10	12 x 1.2 s
SIMPLE	4	4	interstim. interval: 1 s
		**Voice Recognition**	
CHALLENGING	10 old (learned)<br>10 new (impostor)	60	4 x 1.2 s<br>interstim. interval: 0.5 s
SIMPLE	4 old (learned)<br>4 new (impostor)	24	3 trials per voice identity

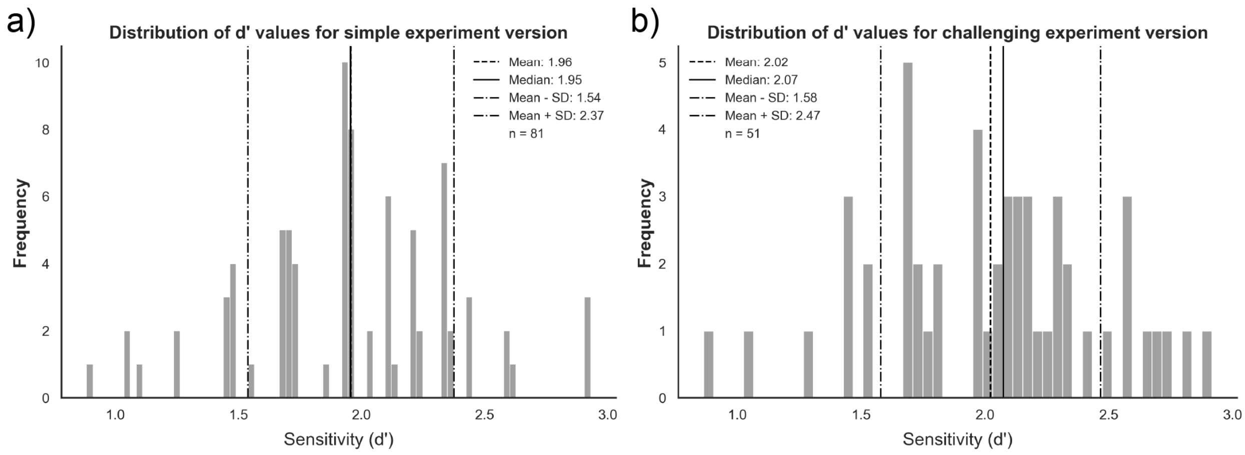

Figure S1. Voice discrimination performance (measured as d') for the simple a) and b) challenging experiment version

Table S2. Pymer interaction estimates

Interaction Parameter	Estimate
Version1:TaskAwareness1	0.658
Version1:Order1	0.209
TaskName1:Order1	0.841
Version1:TaskAwareness1:Order1	0.614

Between-Subjects Analysis (Task 1 Only)

For the between-subjects analysis, we examined the effects of *task awareness* and *task version* on participants' performance, indexed by d'. Performance was analyzed using an ordinary least squares (OLS) linear model with *Task Awareness* (implicit vs. explicit) and *Version* (simple vs. challenging) entered as fixed effects. Statistical inference was performed using a Type II analysis of variance (ANOVA). All analyses were conducted in Python using the `statsmodels` package.

To examine the effect of task awareness and version on d' values, we started by using a model that further included interaction terms (EQ 1):

$$d' \sim \text{Task Awareness} * \text{Version} \tag{1}$$

Given the absence of significant interactions (F(1, 128) = 0.18, p = .67), the interaction term was excluded, and the model was re-estimated with main effects only (EQ 2):

$$d' \sim \text{Task Awareness} + \text{Version} \tag{2}$$

Results from the reduced model:

- Main effect of Task Awareness: $F(1,129) = 3.88$, $p = .05$
- Main effect of Version: $F(1,129) = 5.03$, $p = .03$

The reduced model revealed a main effect of Task Awareness, F(1, 129) = 3.88, p = .05, such that performance was higher in the implicit than in the explicit condition. A significant main effect of Version was also observed, F(1, 129) = 5.03, p = .03, with higher performance in the simple compared to the challenging version.
